# Garlic Organosulfur Compounds Reduce Inflammation and Oxidative Stress during Dengue Virus Infection

**DOI:** 10.3390/v9070159

**Published:** 2017-06-23

**Authors:** Alex Hall, Andrea Troupin, Berlin Londono-Renteria, Tonya M. Colpitts

**Affiliations:** Department of Pathology, Microbiology and Immunology, University of South Carolina School of Medicine, Columbia, SC 29209, USA; arhall@g.clemson.edu (A.H.); Andrea.troupin@uscmed.sc.edu (A.T.)

**Keywords:** dengue virus, inflammation, oxidative stress response, alternative medicine

## Abstract

Dengue virus (DENV) is a mosquito-borne flavivirus that causes significant global human disease and mortality. One approach to develop treatments for DENV infection and the prevention of severe disease is through investigation of natural medicines. Inflammation plays both beneficial and harmful roles during DENV infection. Studies have proposed that the oxidative stress response may be one mechanism responsible for triggering inflammation during DENV infection. Thus, blocking the oxidative stress response could reduce inflammation and the development of severe disease. Garlic has been shown to both reduce inflammation and affect the oxidative stress response. Here, we show that the garlic active compounds diallyl disulfide (DADS), diallyl sulfide (DAS) and alliin reduced inflammation during DENV infection and show that this reduction is due to the effects on the oxidative stress response. These results suggest that garlic could be used as an alternative treatment for DENV infection and for the prevention of severe disease development.

## 1. Introduction

Garlic has been used for centuries for the treatment of medical ailments and human disease [1,2,3,4,5,6]. Crushed garlic contains active organosulfur compounds, which are responsible for the biological activity of garlic. Several of these compounds have been shown to have specific therapeutic and preventative effects on disease development and progression, including alliin, diallyl sulfide (DAS) and diallyl trisulfide (DATS) [2,7]. The combined data from a number of studies also indicates that the active components of garlic may be used to combat inflammation due to a variety of disorders [7].

Dengue virus causes serious human disease and mortality worldwide. In recent years, there has been increased epidemic activity and geographic expansion of dengue infection along with its mosquito vector, and it is considered a serious emerging global health problem [8,9,10,11]. There are no vaccines or specific therapeutic agents approved for dengue virus, aside from Dengvaxia (Sanofi-Paseur, Lyon, France), which has been approved in a few endemic countries [12]. Investigation of novel treatments to alleviate symptoms and lessen disease severity during dengue infection is necessary.

Inflammation is a main component of the host immune response to dengue infection, and can have both protective and pathogenic roles. For example, interferon gamma (IFN-γ), interleukin 12 (IL-12) and interleukin 18 (IL-18) are essential for an effective host immune response against dengue virus infection [13,14]. Other pro-inflammatory cytokines play pathogenic roles during dengue infection. For example, studies have shown that tumor necrosis factor α (TNF-α), IL-8 and IL-10 levels are higher in patients with severe dengue versus mild dengue fever [15,16,17]. Together, data have shown that excessive inflammation and the increased levels of certain cytokines and chemokines can contribute to vascular leakage and endothelial permeability that may lead to severe forms of dengue disease [18,19,20]. Thus, the reduction of inflammation during dengue infection is key to reducing the chance that severe forms of dengue will develop. 

Several herbal supplements have been under investigation in recent years for their ability to reduce inflammation during disease, including flavonoids, carotenoids and plants such as turmeric and garlic. Garlic has been shown to have anti-inflammatory activity in several studies of disease, including cancer and heart disease [7]. Garlic has been shown to have both immunomodulatory and anti-inflammatory effects in several types of cancer [1,2,21]. One study showed that garlic elicits anti-inflammatory and anti-oxidative responses that can stop emerging tumor growth [1]. Another study showed that several organosulfur compounds found in garlic, including DAS, DADS and DATS, were able to destroy cells in a glioblastoma, indicating that garlic may be clinically useful to eradicate brain cancer cells in patients [22,23]. 

The oxidative stress response during dengue virus infection has been linked to the development of inflammation as well as the development of severe disease (dengue hemorrhagic fever (DHF)/dengue shock syndrome (DSS)) [24,25]. It is thought that free radical release during the oxidative stress response may trigger the induction of inflammatory cytokines, leading to excessive inflammation and severe symptoms during dengue infection [26,27]. The results of these studies suggest that blocking the oxidative stress response may reduce the pro-inflammatory response leading to severe symptoms in dengue virus infection. Garlic has been shown to suppress nitric oxide production through the inhibition of NF-κB (nuclear factor kappa-light-chain-enhancer of activated B cells), downregulating expression of inducible nitric oxide synthase (iNOS) and blocking nuclear translocation of NF-κB [28]. Garlic has also been found to inhibit oxidative injury in liver through adenosine monophosphate (AMP)-activated protein kinase [29]. 

Thus, the combined data from a number of studies has shown that garlic has anti-inflammatory activities, including the reduction of pro-inflammatory cytokines such as TNF-α, which is known to be a key player in the development of severe disease in dengue infection. Garlic has also been shown to act through the reduction of oxidative stress, and may inhibit the cytokine-induced inflammation found in severe dengue cases.

Here, we examined the role that garlic has on inflammation and the oxidative stress response during dengue virus infection in vitro.

## 2. Materials and Methods

### 2.1. Cells

The cell lines Huh-7 (kind gift of Brett Lindenbach, Yale University) and U937 (ATCC) were used for all studies. Cells were maintained in DMEM (Huh-7) or RPMI 1640 (U937) complete medium with 10% FBS (fetal bovine serum) at 37 °C with 5% CO_2_.

### 2.2. Quantitative Reverse Transcription (qRT)-PCR

Briefly, total RNA was isolated from cells using RNeasy kit according to manufacturer’s instructions (Qiagen, Valencia, CA, USA). The quantitative reverse transcription (qRT)-PCR assays were conducted using the QuantiFast SYBR Green RT-PCR kit according to manufacturer’s instructions (Qiagen) and a BioRad CFX cycler. Dengue virus primers were: F: 5′-CATTCCAAGTGAGAATCTCTTTGTCA-3′ and R: 5′-CAGATCTCTGATGAATAACCAACG-3′; Viral gene expression was normalized to human β-2 microglobulin using a purchased primer set (#HP207421 Origene, Rockville, MD, USA).

### 2.3. ELISA

Standard ELISAs were performed to measure the levels of IL-8, IL-10, and TNF-α in the cell supernatants. The following kits were used: Hu IL-8 Cytoset (#CHC1303; Invitrogen, Carlsbad, CA, USA), Hu IL-10 Cytoset (#CHC1323; Invitrogen), and Hu TNF-α Cytoset (#CHC1753; Invitrogen). All kits were used according to the manufacturer’s instructions. Briefly, supernatant was diluted and coated onto plates in a 96-well format. Capture (purified) and detection antibodies (Invitrogen) used were: IL-8 (#58.130.09 and #58.130.03), IL-10 (#58.132.09 and #58.132.03), TNF-α (#58.175.09 and #58.175.03). Serially diluted recombinant cytokine standards specific for each cytokine (Invitrogen) were used to generate standard curves. Standard ELISA was also performed to measure levels of iNOS in cell supernatants using capture antibody #ab129372 (Abcam, Cambridge, MA, USA) and HRP detection Ab #7074S (Cell Signaling, Danvers, MA, USA). Plates were incubated with the tetramethylbenzidine (TMB) substrate, BioFX TMB One Component HRP Microwell Substrate (#TMBW-0100-01; SurModics) for 30 min at room temperature (RT). The reaction was stopped with 2 N sulfuric acid and plates were read at 450 nm in a BioTek Synergy HT microplate reader (Biotek Instruments, Winooski, VT, USA) and Gen5 Data Analysis Software (Biotek). 

### 2.4. Dengue Virus

We used DENV-2 NGC (New Guinea C) virus for these studies. Virus was propagated in C6/36 *Aedes albopictus* mosquito cells and titered by plaque assay using the Vero monkey kidney cell line [30]. Virus was added to either Huh7 or U937 cells at an MOI (multiplicity of infection) of 1.0 for all studies unless otherwise indicated. Briefly, virus was added to cell cultures and allowed to infect for 1 h at 37 °C. The cells were washed, and infection allowed to continue for indicated times. Cell lysates and supernatants were used at 24 h post-infection.

### 2.5. Garlic Treatment

We used three active organosulfur garlic compounds in our assays: DAS (#103021000; Acros Organics, Geel, Belgium), DADS (#A14400; Pfaltz & Bauer, Waterbury, CT, USA) and Alliin (#027253S; Indofine Chemical Company Inc., Hillsborough Township, NJ, USA). Cells were treated with varying concentrations of garlic compounds, as indicated in the figure legends. 

### 2.6. Lipid Peroxidation Assay

We measured malondialdehyde (MDA) using the Lipid Peroxidation (MDA) Assay kit (#ab118970; Abcam) to fluorometrically measure lipid peroxidation according to manufacturer’s instructions. The colorimetric reaction was measured at 532 nm on a BioTek Synergy HT microplate reader (Biotek Instruments) and Gen5 Data Analysis Software (Biotek).

### 2.7. Western Blot

Cell lysates were run on a 4–12% SDS-PAGE (sodium dodecyl sulfate polyacrylamide gel electrophoresis) gel (Bio-Rad, Hercules, CA, USA) and the proteins were transferred to a nitrocellulose membrane. The membrane was blocked with 5% milk in 1% TBST (Tween-20 tris-buffered saline) for 1 h at RT and then incubated with a primary antibody specific for iNOS (ab129372; Abcam) overnight at 4 °C. The membrane was washed and then incubated with the appropriate horseradish peroxidase secondary antibody for 1 h at RT. The protein blots were incubated with ECL substrates (Bio-Rad) for 5 min at RT and then detected on CL-Xposure Film (Thermo Scientific, Skokie, IL, USA) and developed.

## 3. Results

### 3.1. Garlic Organosulfur Compounds Reduce Inflammation during Dengue Virus Infection

**Figure 1:**
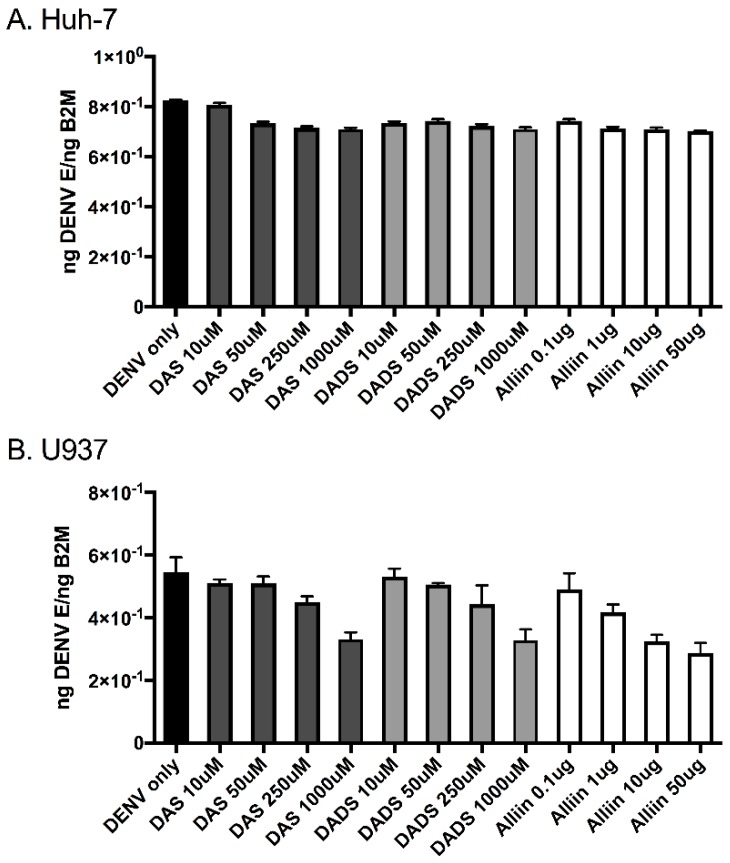
Garlic organosulfur compounds do not significantly affect dengue virus infection. Cells were treated with indicated doses of garlic organosulfur compounds and immediately infected with dengue virus 2 New Guinea C (DENV 2 NGC) at multiplicity of infection (MOI) of 1.0. At 24 h post-infection, RNA was isolated from cell lysates and used in quantitative reverse transcription (qRT)-PCR analysis to detect dengue virus infection levels. Viral gene expression was normalized to human β-2 microglobulin expression. (**A**). Huh-7 cells; (**B**). U937 cells. Data is expressed as mean ± SD. Technical and biological replicates were done in triplicate. DAS: diallyl sulfide; DADS: diallyl disulfide.

**Figure 2:**
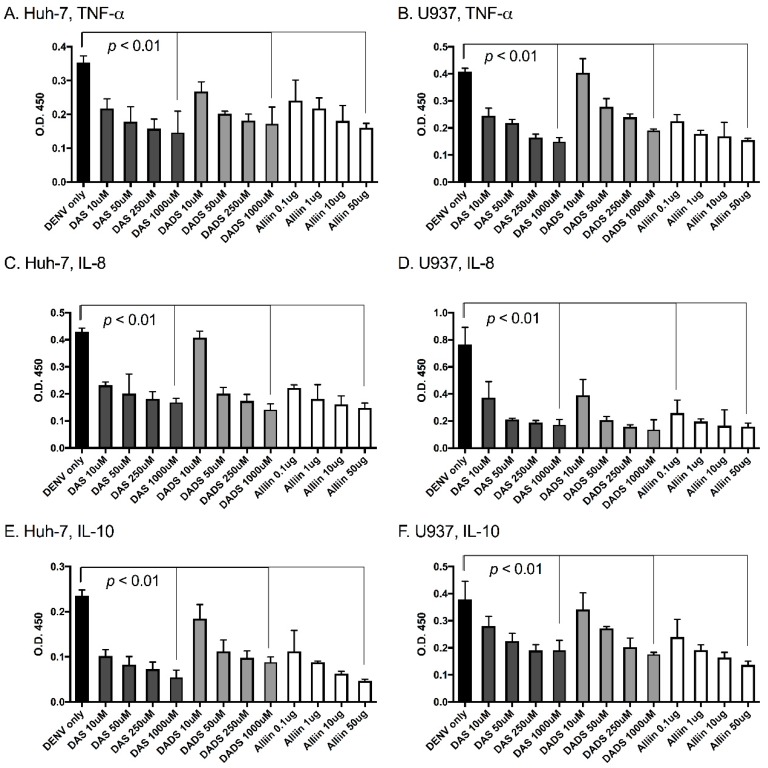
Garlic organosulfur compounds significantly reduced inflammatory cytokines during dengue virus infection. Cells were treated with indicated doses of garlic organosulfur compounds and infected with DENV2 NGC at MOI of 1.0. At 24 h post-infection, cell supernatant was removed and analyzed by ELISA for levels of pro-inflammatory cytokines. Cells and cytokine are as follows: (**A**). Huh-7 cells-tumor necrosis factor α (TNF-α); (**B**). U937 cells-TNF-α; (**C**). Huh-7 cells-interleukin (IL)-8; (**D**). U937 cells-IL-8; (**E**). Huh-7 cells-IL-10; (**F**). U937 cells-IL-10. Technical and biological replicates were done in triplicate. ANOVA and Mann-Whitney were used to calculate statistical significance.

To investigate the effects of garlic on inflammation during dengue virus infection, we used three active organosulfur garlic compounds: DAS, DADS and Alliin. We chose our doses based on published literature [7]. We chose to use two human cell lines in our study due to the cells’ involvement in natural dengue virus infection: U937 human macrophage-like cells and Huh-7 human liver cells. First, we examined dengue virus infection levels with and without the presence of garlic compounds. We found that the addition of the compounds did not significantly affect dengue virus infection levels in the cells (Figure 1A,B). This was somewhat expected and removed the variable of changing infection levels from subsequent studies. Next, we examined the levels of the pro-inflammatory cytokines TNF-α, IL-8 and IL-10 in the supernatants of dengue virus-infected cells with and without the garlic compounds. Garlic compounds have previously been shown to reduce inflammation during cancer and heart disease [1,7,31]. We found that the addition of garlic compounds reduced the levels of all three pro-inflammatory cytokines during dengue virus infection at all doses examined (Figure 2A–F). 

### 3.2. Garlic Organosulfur Compounds Reduce Oxidative Stress during Dengue Virus Infection

**Figure 3:**
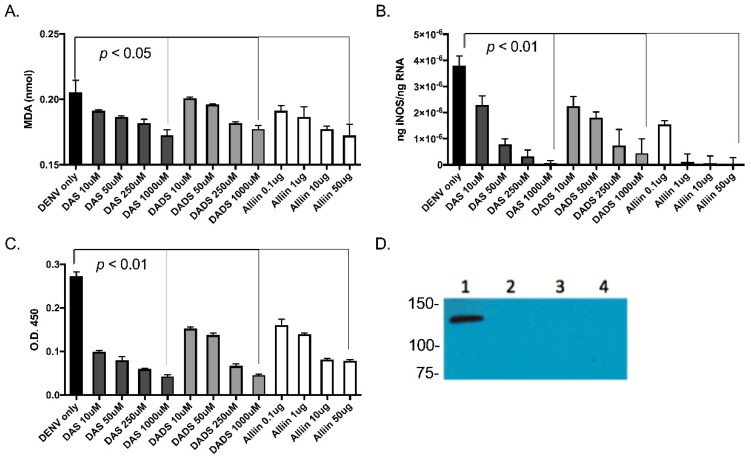
Garlic organosulfur compounds reduced cellular lipid peroxidation, inducible nitric oxide synthase (iNOS) gene expression and both intra- and extracellular protein levels during dengue virus infection. U937 cells were treated with indicated doses of garlic organosulfur compounds and infected with DENV2 NGC at MOI of 1.0. At 24 h post-infection, cell lysates were used to measure malondialdehyde (MDA) as an indication of lipid peroxidation (**A**). RNA was isolated from cell lysates and used in qRT-PCR analysis to detect levels of intracellular iNOS mRNA (**B**). Cell supernatant was used in ELISA analysis to measure secreted and extracellular levels of iNOS protein (**C**). Cell lysate was also used to measure intracellular protein levels of iNOS via Western blot analysis (**D**). Western blot lanes: 1. No garlic compounds (Control); 2. 1000 uM DAS 3. 1000 µM DADS; 4. 50 µg Alliin. Equivalent protein amounts were loaded into each lane as an internal control. Technical and biological replicates were done in triplicate.

As oxidative stress is thought to be the mechanism through which dengue virus acts to trigger the pro-inflammatory immune response during infection, and garlic has previously been shown to suppress nitric oxide production [28], inhibit oxidative injury [29] and decrease reactive oxygen species (ROS) levels [32], we examined the anti-oxidant activity of garlic organosulfur compounds during dengue virus infection. Oxidative stress induced by lipid peroxidation has been shown to contribute to inflammation. We planned to quantify lipid peroxidation as a measure of oxidative stress. The end products of lipid peroxidation are reactive aldehydes such MDA and 4-hydroxynonenal (4-HNE), and measuring these end products is a widely accepted assay for oxidative damage. To this end, we measured MDA using the Lipid Peroxidation (MDA) Assay kit (Abcam) to fluorometrically measure lipid peroxidation in dengue virus-infected cells. We found that with higher doses of garlic compounds, levels of MDA produced from the infected cells were reduced (Figure 3A). 

Levels of iNOS have been correlated with increased inflammation in several infection models and can be a marker for the oxidative stress response [33]. There is also evidence for an association between increased vascular nitric oxide and severe dengue disease [34]. To this end, we measured levels of iNOS in the U937 cells that we infected with dengue virus with and without garlic. We first isolated RNA from the cells and tissues and quantified iNOS using qRT-PCR analysis (Figure 3B). We next quantified iNOS protein levels in cell lysates using antibodies with ELISA (Figure 3C) and Western blot analysis (Figure 3D). Our results indicate that levels of iNOS were reduced with the addition of active garlic compounds during dengue virus infection.

In summary, we found that garlic organosulfur compounds acted to reduce inflammation and oxidative stress during dengue virus infection in vitro.

## 4. Discussion

Dengue virus infection has an enormous impact on the health and economies of tropical and subtropical regions, with dengue infections occurring in Asia, the Americas, Africa, Pacific and Mediterranean regions [11,35,36,37,38]. In addition, there have been recent outbreaks in Texas and Florida where transmission occurred on American soil [39,40]. As there are no specific antiviral regimens for the treatment of dengue virus infection, or the development of severe disease [41], it is imperative to investigate alternative treatments, including naturally bioactive compounds and herbal supplements.

Inflammation during dengue virus infection plays both beneficial and pathogenic roles. Studies in mice have shown that TNF-α levels are linked to increased vascular leakage during dengue infection and that blocking TNF-α results in reduced lethality due to dengue infection [42,43,44]. Levels of another pro-inflammatory cytokine, macrophage inhibitory factor (MIF), have also been correlated with severe dengue infection in humans [45], and MIF knock-out mice develop less severe dengue infection and have higher survival than wild-type mice [46]. Various chemokines have also been associated with dengue severity and clinical outcomes in humans. Levels of the chemokine C-C ligands 2, 3 and 4 (CCL2, CCL3 and CCL4) have been correlated with severe dengue disease in humans in several studies [47,48], and mice lacking these chemokines have reduced lethality and milder disease during dengue infection [49]. Thus, the reduction of inflammation during dengue virus infection could reduce the length and severity of disease.

Garlic has been shown to have several health benefits, and many of these are thought to be due to the reduction of inflammation. The bioactive compounds from garlic have been shown to have anti-inflammatory activity during cancer and heart disease [7]. Garlic has been shown to have both immunomodulatory and anti-inflammatory effects in several types of cancer [1,2,21]. One study showed that garlic elicits anti-inflammatory and anti-oxidative responses that can stop emerging tumor growth [1]. Another study showed that several organosulfur compounds found in garlic, including DAS, DADS and DATS, were able to destroy cells in a glioblastoma, indicating that garlic may be clinically useful to eradicate brain cancer cells in patients [22,23]. Further investigation is needed to gain a comprehensive picture of how garlic reduces inflammation, and in what disease states this can be used as treatment.

The development of inflammation during severe forms of dengue has been associated with the oxidative stress response, which can trigger the production of inflammatory cytokines [24,25,26,27]. In addition, lipid peroxidation and an increase in redox status as measured by MDA levels have also been linked with an increase in pro-inflammatory cytokines in patients with severe dengue disease [27]. It has been shown that an organosulfuric compound found in garlic has the ability to inhibit TNF-α-induced inflammation by decreasing ROS levels [32]. Garlic has also been shown to act as an anti-oxidant in liver cells by inducing the antioxidant enzyme glutamate-cysteine ligase, which increases glutathione (GSH) content and prevented hydrogen peroxide production and cell death [5]. 

In this study, we found that garlic organosulfur compounds were able to reduce both inflammation and oxidative stress during dengue virus infection of human cells. This could lead to a novel alternative therapeutic for treatment of infection and/or prevention of progression to severe disease. We next plan to examine the effects of garlic consumption on dengue virus infection in a human patient cohort.
